# Effect of Fungi on Metabolite Changes in Kimchi During Fermentation

**DOI:** 10.3390/molecules25215040

**Published:** 2020-10-30

**Authors:** Seung-Ho Seo, Seong-Eun Park, Eun-Ju Kim, Kwang-Moon Cho, Sun Jae Kwon, Hong-Seok Son

**Affiliations:** 1 School of Korean Medicine, Dongshin University, Naju, Jeonnam 58245, Korea; blue784300@naver.com (S.-H.S.); seong9525@naver.com (S.-E.P.); yci3431@naver.com (E.-J.K.); 2 AccuGene Inc., Incheon 22006, Korea; kmcho@accugenelab.co.kr

**Keywords:** kimchi, fungi, fermentation, GC-MS, metabolomics

## Abstract

The purpose of this study is to investigate the effect of fungi on kimchi metabolites during fermentation. A gas chromatography-mass spectrometry (GC-MS) based metabolite profiling approach in combination with principal component analysis (PCA) is performed to differentiate metabolites produced by fungi or bacteria. To avoid bacterial growth, kimchi is treated with 100 μg/mL of ampicillin every three days from 30 to 50 days of fermentation. The relative content of the major fungi at 50 days of fermentation, between the control group and the ampicillin treatment group, was not significantly different. The administration of ampicillin changed the metabolites in kimchi by affecting the growth of kimchi bacteria. Based on the pattern of change of each metabolite, the changed metabolites are grouped into four categories: (1) metabolites produced or consumed by fungi, (2) metabolites involving both fungi and bacteria, (3) metabolites produced or consumed by bacteria, and (4) metabolites of undetermined origin. Alanine, thymine, galacturonic acid, and malonic acid can be regarded as the metabolites produced by fungi between 30 and 50 days of fermentation. In contrast, malic acid, oxaloacetic acid, galactitol, glucose, and mannitol are presumed to be the metabolites mainly consumed by fungi. This study is meaningful as the first study conducted by inhibiting growth of bacteria to identify the metabolites contributed by fungi or bacteria in the kimchi fermentation process. These results could be used to make customized kimchi that controls the production of desired metabolites by selectively controlling the formation of microbial communities in the kimchi industry.

## 1. Introduction

Kimchi is a traditional Korean cuisine made from kimchi cabbage and various seasonings including ginger, garlic, red pepper, spring onion, jeotgal (salted seafood), and salts [1]. There are many types of kimchi in Korea made with various vegetables as the main ingredients. In order to eat fresh vegetables that are not available in winter, there is a culture called ‘gimjang’ in Korea, which manufactures kimchi that can be consumed for a long time (more than a year) [2]. In general, kimchi made in early winter is stored at low temperatures and is consumed until the next year.

Spontaneous fermentation in kimchi leads to the formation of various microorganisms, affecting the sensory qualities of kimchi [3]. It is well known that the composition of metabolites important to kimchi taste and flavor, such as organic acids (lactic acid and acetic acid) and other flavoring compounds (mannitol and amino acids), are directly affected by the kimchi microbial community [4]. The microorganisms essential for kimchi production can be divided into lactic acid bacteria (LAB) and yeast. LAB are formed in the early stages of kimchi fermentation, whereas yeast is known to be formed in the middle stages of kimchi fermentation [5,6]. In many previous studies, LAB have been primarily studied as the key microorganisms responsible for kimchi fermentation [3,7,8,9,10]. Yeast, known as a symbolic microorganism in many fermented foods due to the production of alcoholic compounds and carbonic acid [11,12,13,14], can also significantly affect the quality of kimchi. However, the changes in the metabolites by yeast during fermentation and storage of kimchi have not been studied.

Metabolomics has been successfully applied to monitor metabolic changes caused by microorganisms during fermentation [15]. Recently, the combination of metabolomics and amplicon sequencing has been found to be a very comprehensive approach for investigating the relationship between microbial communities and metabolites [16,17,18,19,20]. Many studies have actively attempted to interpret changes in kimchi metabolites according to salinity [19,21] and starter culture [4,20] in connection with the kimchi microbial community. However, these kimchi studies only dealt with bacterial microbial communities and no studies have identified metabolic changes according to yeast microbial communities. Until now, there have been no studies on the sequencing of the internal transcribed spacer (ITS) region in kimchi, so nothing is known about the fungal or yeast community contained in kimchi.

Therefore, this study was conducted to investigate the effect of the fungal community, including yeast, on kimchi metabolites. After killing bacteria during kimchi fermentation, the effects of fungi on kimchi metabolites were investigated by comparing the metabolic differences compared to the control group. This study is meaningful as the first study to identify metabolites contributed by fungi or bacteria in the fermentation process of kimchi.

## 2. Results

### 2.1. Fungal Community Change during Kimchi Fermentation

**Figure 1:**
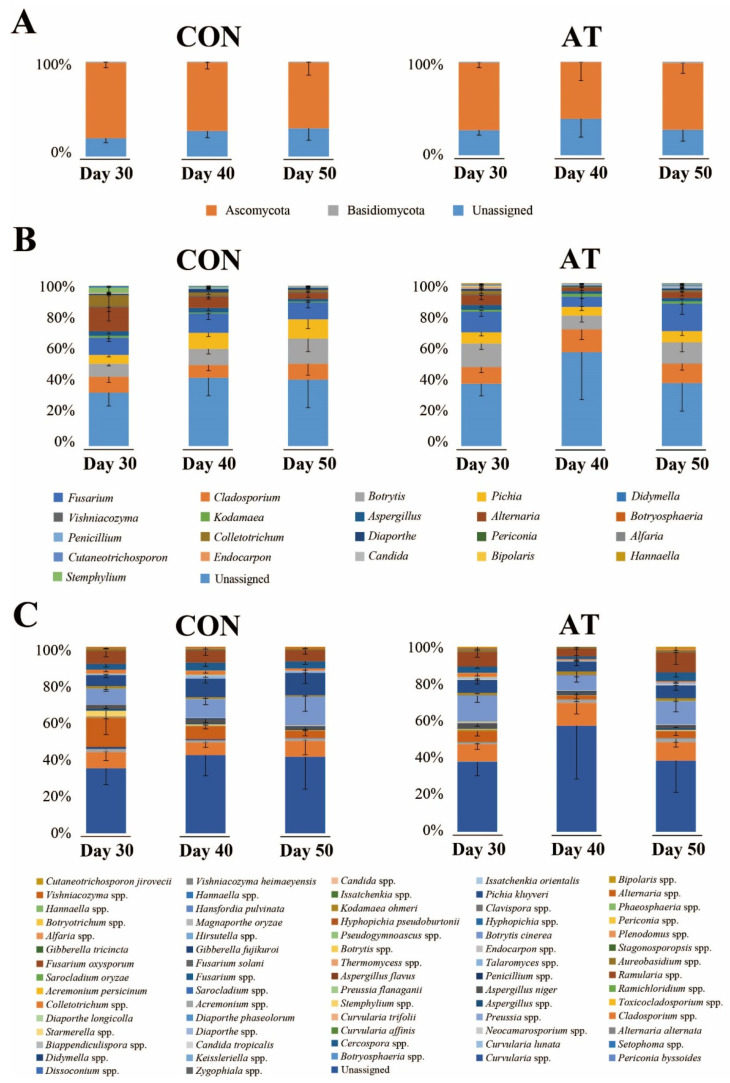
The kimchi fungal community profiles at the phylum (**A**), genus (**B**), and species (**C**) levels in the control group (CON) and the ampicillin-treated group (AT) during 30 to 50 days of fermentation as revealed by internal transcribed spacer 2 (ITS2) sequencing. The x-axis represents different samples. The y-axis represents the percentage abundance of fungus in each sample.

Figure 1 shows the results of the microbial community analysis between experimental groups at the phylum, genus, and species levels. The dominant phylum across the entire eukaryotic population was Ascomycota (72.70%), although it also contained Basidiomycota (1.12%) and unidentified fungal microorganisms (25.89%), on the 30th day of fermentation (Figure 1A). At the genus level, the dominant genera were *Cladosporium*, *Fusarium*, *Pichia*, *Botrytis*, and *Alternaria* (Figure 1B). The relative content of the five major genera (cutoff > 5%) at 50 days of fermentation between the control group (CON50) and the ampicillin-treated group (AT50) were not significantly different (data not shown, *p* < 0.05, Mann-Whitney U test), indicating that the fungal community was not significantly affected by ampicillin treatment. On the 30th day of fermentation, the dominant genera identified at the species level were *Botrytis cinerea* (4.64%), *Fusarium oxysporum* (3.87%), *Pichia kluyveri* (3.21%), *Aspergillus niger* (0.96%), and *Kodamaea ohmeri* (0.71%) (Figure 1C).

### 2.2. Metabolic Change during Kimchi Fermentation

**Figure 2:**
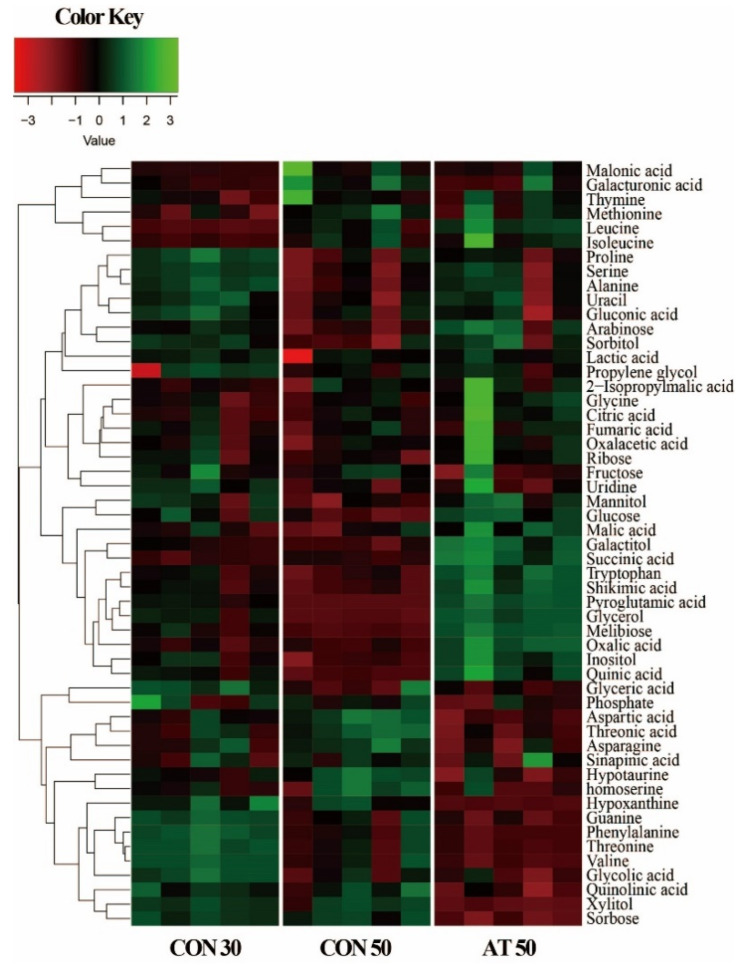
A heatmap showing metabolite differences between the control groups (CON30 and CON50) and the ampicillin-treated group (AT50). CON30 and CON50 refer to the control group on the 30th and 50th day of fermentation, respectively. AT50 refers to the ampicillin-treatment group on the 50th day of fermentation.

In this study, a total of 53 metabolites were identified in kimchi samples by gas chromatography-mass spectrometry (GC-MS) analysis. Variation in the identified metabolites for each group was depicted in a heatmap (Figure 2). As expected, some metabolite levels in kimchi were markedly changed according to the fermentation period. In a comparison between samples on the 50th day of fermentation, kimchi treated with ampicillin showed a metabolite pattern different from the control.

**Figure 3:**
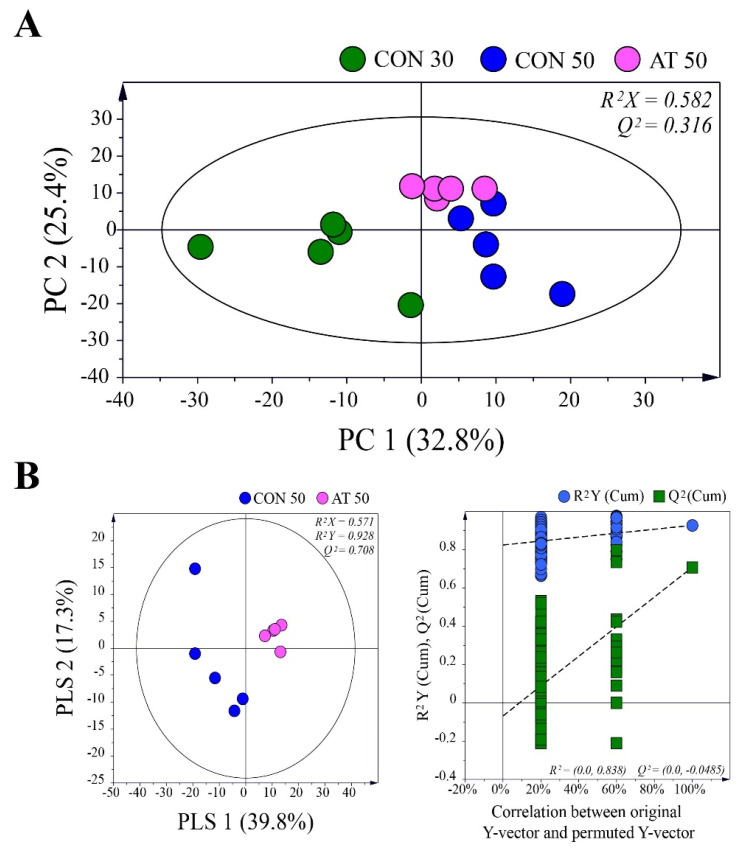
(**A**) Principal component analysis (PCA) score plots derived from gas chromatography-mass spectrometry (GC-MS) data for control groups (CON30 and CON50) and the ampicillin-treated group (AT50) during kimchi fermentation. (**B**) Partial least squares-discriminant analysis (PLS-DA) score plot between the CON50 and AT50 groups after 50 days of fermentation validated by a permutation test.

To investigate metabolic changes in the kimchi samples during fermentation by the administration of ampicillin, principal component analysis (PCA) was performed using GC-MS data for control groups (CON30 and CON50) and the ampicillin-treated group (AT50) (Figure 3A). A clear separation of samples between day 30 and day 50 was observed in the PCA score plot, indicating that the metabolic profile was changed by the fermentation period. On the 50th day of fermentation, kimchi samples were also separated by the administration of ampicillin, suggesting that the administration of ampicillin changed the metabolites in kimchi by affecting the growth of kimchi bacteria.

The partial least squares-discriminant analysis (PLS-DA) method was used to refine the separation between the CON50 and AT50 groups already established via PCA (Figure 3B). No over-fitting was observed in the cross-validation performed using 200 repeated permutation tests. According to the criterion of variable importance in projection (VIP) > 1.0 and *p* < 0.05, a total of 25 metabolites were identified as metabolites contributing to the differential PLS-DA model between the groups. Table 1 shows the difference in metabolites in each group. A false discovery rate was applied to all tests to correct for multiple testing. Based on the pattern of change of each metabolite, they were largely grouped into the following four categories: (1) metabolites produced or consumed by fungi, (2) metabolites involving both fungi and bacteria, (3) metabolites produced or consumed by bacteria, and (4) metabolites of undetermined origin.

## 3. Discussion

Since household kimchi manufactured in Korea does not use a starter culture, it is fermented through various microorganisms derived from the raw materials [22]. LAB are formed from the beginning of kimchi fermentation, whereas yeast is known to appear in the middle and late stages of fermentation [23]. The organic acids or antimicrobial compounds produced by LAB inhibit yeast growth, making it difficult for yeast to grow during kimchi fermentation [24,25,26]. According to Jeong et al. [27], the abundance of bacteria and yeast showed a negative correlation during kimchi fermentation, suggesting that there may be antagonism between bacteria and yeast. The appearance time of yeast and the number of viable cells differ depending on the kimchi manufacturing conditions. Lee et al. [5] reported that yeast began to be detected in kimchi from the 10th week of storage and reached 5.91 log CFU/g on the 20th week. According to the results of Jeong et al. [6], yeast increased from 19 days of fermentation with a decrease in bacteria, reaching a maximum value of about 7 log CFU/mL at 45 days of fermentation. It is predicted that various fungal communities, including yeast, are involved in the fermentation of kimchi but there have been no studies analyzing the fungal communities.

To the best of our knowledge, this is the first study to investigate fungal community in kimchi. Fungal community present in kimchi can contribute or adversely affect kimchi quality, such as texture, taste, food safety, nutrition, and functionality. Fai et al. [28] reported that *Pichia kluyveri* strain showed to be the best candidate for probiotic use among the yeasts tested, taking into account properties such as colonization and survival in the digestive tract, and the ability of antagonism against enteropathogenic bacteria. In the current study, *Aspergillus niger, Aspergillus flavus,* and *Fusarium*, known to produce mycotoxins associated with multiple human and animal diseases, were also detected in kimchi. There is no research on the mycotoxins contained in kimchi. However, since the main ingredient of kimchi is red pepper powder that is sensitive to mycotoxins contamination [29], the possibility of the presence of mycotoxins in kimchi cannot be excluded. Kimchi can be considered generally safe because it has been consumed safely for a long time. However, kimchi products manufactured by spontaneous fermentation can be exposed to various sources of contaminants, including pathogens, during the manufacturing process. Therefore, the Korea Food and Drug Administration adapted Hazard Analysis Critical Control Point rules to ensure that the hygiene of commercially manufactured kimchi is acceptably safe [30].

In the case of kimchi fermented under similar conditions, the number of viable yeast increases rapidly between 30 and 50 days of fermentation [6]. In the current study, to identify the metabolites produced or consumed by yeast during kimchi fermentation, kimchi was treated with ampicillin to selectively kill only bacteria. Ampicillin is known to inhibit bacterial cell wall synthesis through binding to penicillin-binding proteins [31]. Among the metabolites that changed significantly during 30 and 50 days of fermentation (Table 1), those that were not affected by treatment with ampicillin are presumed to be the metabolites related to fungi. Although alanine, thymine, galacturonic acid, and malonic acid increased in both the CON50 and AT50 groups compared to the CON30 group, there was no significant difference between the CON50 and AT50 groups. Therefore, these metabolites can be regarded as the metabolites produced by fungi between 30 and 50 days of fermentation. Likewise, malic acid, oxaloacetic acid, galactitol, glucose, and mannitol are presumed to be the metabolites mainly consumed by fungi. Among them, galacturonic acid [32,33], glucose, and mannitol [27] have been reported to be correlated with *Pichia* and *Candida* in other kimchi studies. Both yeasts were also detected in this study. Interestingly, a reduction in the sugar alcohols (galactitol and mannitol) was observed. Mannitol is known to be an important component providing a refreshing taste to kimchi. Mannitol can be produced by LAB using fructose as an electron acceptor [34]. According to a kimchi study using *Leuconstoc mesenteroides* as a starter, mannitol increased between 10 and 30 days of kimchi fermentation [4]. Jeong et al. [27] reported that the concentration of mannitol increased until 30 days of fermentation, and then gradually decreased, which was presumed to be due to metabolism by yeast. However, it is very difficult to identify which fungi species of kimchi are mainly involved in the production of metabolites.

In contrast, citric acid, fructose, uridine, and ribose decreased in both the CON50 and AT50 groups compared to the CON30 group, but the AT50 group was significantly higher than the CON50 group. These metabolites can be considered the metabolites consumed by both bacteria and fungi. Similarly, it is presumed that both bacteria and fungi were involved in the production of propylene glycol during kimchi fermentation. The metabolites with significant increases or decreases in the CON50 group but no significant difference from the AT50 group were lactic acid, gluconic acid, sinapinic acid, fumaric acid, and tryptophan. These metabolites indicate that bacteria were mainly produced or consumed during the kimchi fermentation. There was no change in the CON50 group, but significant increases and decreases in several amino acids were observed in the AT50 group. It is presumed that these metabolites were caused by changes in the microbial ecosystem in kimchi caused by ampicillin treatment.

Recently, studies have attempted to understand the relationship between microbial communities and metabolites through a combination of metabolomics and amplicon sequencing. For example, Kim et al. [35] revealed that the metabolic profile of kimchi was not significantly affected by the difference in white colony-forming yeast diversity using high-throughput DNA sequencing and metabolomics techniques. Most of the studies that focused on the fungi of kimchi, not yeast, dealt with the safety of kimchi [36,37,38]. Therefore, this study is meaningful as the first study conducted by killing bacteria with ampicillin to determine the metabolites contributed by fungi in the kimchi fermentation process. However, the changes in the bacterial ecosystem of kimchi caused by ampicillin may affect the microbial growth environment of the fungi, so the results of the metabolites derived from this study may differ from those in kimchi. To determine which fungi actually produce or consume metabolites during kimchi fermentation, it is necessary to analyze changes by selectively adding individual strains.

## 4. Materials and Methods 

### 4.1. Kimchi Preparation and Antibiotic Treatment

Kimchi was prepared by modifying the method of the World Institute of Kimchi (Gwangju, Korea) [39]. Briefly, a seasoning mixture was prepared by mixing jeotgal, garlic, ginger, onion, radish, glutinous rice porridge, red pepper powder, and water in the weight ratio of 70:3.75:1.2:1.85:2.4:4.5:1.2:4.5:2:2:6.6. This seasoning mixture was added to kimchi cabbage at a ratio of 9:1 (*w*/*w*). The final salt concentration in kimchi was adjusted to 2.5%. This kimchi mixture was homogenized using a blender and then placed in a 20 kg plastic container. The kimchi was placed at room temperature for 24 h for active fermentation and then stored at 4 °C for 30 days. On the 30th day of fermentation, the kimchi was divided into 10 batches of 2 kg each. To prevent bacterial growth, 100 μg/mL of ampicillin [40] was added to five batches of kimchi every three days from 30 to 50 days of fermentation. According to the results of antibiotic susceptibility testing for *Lactobacillus* and *Bifidobacterium* [41], the minimum inhibitory concentration of ampicillin was 1 μg/mL or less for all tested strains. During the experimental period, bacteria were cultured using de Man, Rogasa and Sharpe (MRS) broth every three days in the kimchi samples, but no viable cells were detected. Kimchi samples were taken at 30, 40, and 50 days of fermentation for analyses. CON30, CON40, and CON50 refer to the control group on the 30th, 40th and 50th day of kimchi fermentation. AT30, AT40, and AT50 refer to the ampicillin-treatment group on the 30th, 40th and 50th day of kimchi fermentation. The supernatants were separated from the kimchi by centrifugation (4 °C, 10 min, 12,000 rpm) and stored at −80 °C prior to microbial community and metabolite analyses. The research overall design and flow process used in this study are depicted in Figure S1.

### 4.2. DNA Extraction and ITS2 Sequencing

DNA was extracted using an AccuFAST automation system (AccuGene, Incheon, Korea) according to the manufacturer’s instructions. The internal transcribed spacer 2 (ITS2) regions were amplified from the DNA extracts through 25 PCR cycles using KAPA HiFi HotStart ReadyMix (Roche, Basel, Switzerland) and fusion primers fITS9/ITS4 [42] containing Nextera adaptor sequences. The PCR products were purified with HiAccuBeads (AccuGene). The amplicon libraries were pooled at an equimolar ratio and the pooled libraries were sequenced on an Illumina MiSeq system using a MiSeq Reagent Kit v2 for 500 cycles (Illumina, San Diego, CA, USA).

For all raw data sets, VSEARCH v2.10.3 was used to remove chimeric PCR products from the filtered reads [43]. Downstream analyses of the quality and chimera filters were performed using the QIIME 1.9.2 software package [44]. Each of the quality-filtered sequencing read datasets was assigned to operational taxonomic units (OTUs) with a threshold of 97% pairwise identity using QIIME´s reference-based workflow scripts and a UNITE reference database [45].

### 4.3. Metabolite Analysis and Data Processing

The sample preparation methods for metabolite analysis were similar to those described in a previous study [46]. Briefly, after centrifugation (13,000 rpm, 4 °C, 15 min), 50 μL of supernatant was pooled in 1.5 mL Eppendorf tubes. Then, 10 μL of ribitol solution (0.5 mg/L) was added as an internal standard (IS). After freeze-drying, *O*-methoxyamine hydrochloride (20 mg/mL) was added to each sample and incubated (75 rpm, 30 °C, 90 min) in the dark. Silylation was performed by adding 50 μL of *N*-methyl-*N*-trimethylsilyl-trifluoroacetamide. Each sample was vortex mixed, shaken (75 rpm), and incubated at 37 °C for 30 min. After centrifuging the sample at 13,000 rpm for 10 min, GC-MS analysis was performed on the supernatant. To validate the stability and performance of the GC-MS analysis, a quality control (QC) sample was prepared by pooling equal volumes (10 μL) of kimchi from each sample. QC samples were analyzed every 5 samples during the run. Generally, the relative standard deviation (RSD) of each metabolite across the QC samples was used as an indicator of the reproducibility and the repeatability of the analysis. In this study, the average RSD of the QC samples was 4.65%. The signal features used in this study were filtered using RSD values below 30% in QC samples.

The derivatized samples were analyzed using a QP 2020 GC-MS (Shimadzu, Kyoto, Japan). The GC oven temperature was initially held at 80 °C for 2 min and finally increased to 330 °C at a rate of 15 °C/min and held for 6 min. The m/z range was set to 50–600 with electron impact ionization (70 eV). The sample was injected in the split mode (1:50).

The raw GC-MS data were converted into a netCDF format and processed with MetAlign software for peak detection, noise removal, normalization, and alignment [47]. The resulting data was imported into AIoutput software for peak identification and prediction [46]. The identification of metabolites was performed by comparing the mass spectrum to the AIoutput software library, the NIST 14.0 library, and the human metabolome database (HMDB, http://www.hmdb.ca). Multivariate analysis, such as principal component analysis (PCA), was conducted using SIMCA-P version 14.0 software package (Umetrics, Umea, Sweden). Cross-validation was performed using a permutation test (*n* = 200). Metabolites with variable importance in projection (VIP) scores greater than 1.0 and *p*-values lower than 0.05 were considered metabolites capable of discriminating groups.

### 4.4. Availability of Data

The ITS2 gene amplicon datasets presented in this study were deposited in National Center for Biotechnology Information (NCBI) under accession numbers PRJNA670060.

## 5. Conclusions

Although many kimchi studies report changes in metabolites during fermentation, they are interpreted in relation to bacteria, such as LAB. Therefore, this study is meaningful as the first study conducted by inhibiting the growth of bacteria to identify the metabolites contributed by fungi or bacteria in the kimchi fermentation process. The administration of ampicillin changed the metabolites in kimchi by not affecting the relative content of the major fungi. Based on the pattern of change of each metabolite, the changed metabolites were grouped into four categories: (1) metabolites produced or consumed by fungi, (2) metabolites involving both fungi and bacteria, (3) metabolites produced or consumed by bacteria, and (4) metabolites of undetermined origin. These results could be used to make customized kimchi by selectively controlling the formation of microbial communities in the kimchi industry. 

## Figures and Tables

**Table 1:** Significantly different metabolites between the control and ampicillin-treated group after 50 days of kimchi fermentation.

Metabolite	CON30 ^1^	CON50		AT50		AT50/CON50		Related Microorganisms
	Intensity	Intensity	↑/↓ ^2^	Intensity	↑/↓	Fold Change	↑/↓	
Alanine	0.004	0.017	↑ *,^3^	0.023	↑ **	1.353	NS ^4^	Fungi
Thymine	0.012	0.015	↑ *	0.015	↑ **	1.000	NS	
Galacturonic acid	0.119	0.247	↑ **	0.257	↑ ***	1.040	NS	
Malonic acid	0.028	0.062	↑ ***	0.065	↑ ***	1.048	NS	
Malic acid	0.022	0.010	↓ **	0.009	↓ ***	0.900	NS	
Oxaloacetic acid	0.009	0.005	↓ ***	0.006	↓ ***	1.200	NS	
Galactitol	0.097	0.044	↓ ***	0.056	↓ ***	1.273	NS	
Glucose	10.979	8.973	↓ ***	9.617	↓ **	1.072	NS	
Mannitol	2.661	1.861	↓ ***	2.134	↓ **	1.147	NS	
Propylene glycol	0.009	0.012	↑ ***	0.010	↑ *	0.880	↓ *	Fungi + Bacteria
Citric acid	1.080	0.400	↓ **	0.756	↓ **	1.890	↑ **	
Fructose	10.103	4.179	↓ ***	7.538	↓ ***	1.804	↑ **	
Uridine	0.037	0.000	↓ ***	0.019	↓ **	975.157	↑ **	
Ribose	0.415	0.138	↓ ***	0.246	↓ *	1.779	↑ *	
Lactic acid	8.386	9.553	↑ **	8.877	NS	0.929	↓ *	Bacteria
Gluconic acid	0.015	0.020	↑ *	0.017	NS	0.853	↓ *	
Sinapinic acid	0.005	0.003	↓ *	0.004	NS	1.456	↑ **	
Fumaric acid	0.055	0.039	↓ ***	0.048	NS	1.215	↑ *	
Tryptophan	0.003	0.001	↓ ***	0.003	NS	1.814	↑ *	
Leucine	0.510	0.739	NS	1.042	↑ ***	1.411	↑ *	Undetermined
Isoleucine	0.415	0.522	NS	0.695	↑ ***	1.333	↑ *	
Proline	1.304	1.896	NS	2.974	↑ ***	1.568	↑ *	
Methionine	0.088	0.094	NS	0.117	↑ **	1.247	↑ **	
Phenylalanine	0.241	0.224	NS	0.289	↑ *	1.288	↑ **	
Valine	0.026	0.017	NS	0.005	↓**	0.315	↓*	

^1^ CON30, control kimchi at 30 days of fermentation; CON50, control kimchi at 50 days of fermentation; AT50, ampicillin-treated kimchi at 50 days of fermentation. ^2^ The vertical arrows (↓ and ↑) represent a decrease or increase in metabolite levels after 50 days of kimchi fermentation. ^3^ Symbols (*) indicate significant differences (*, *p* < 0.05; **, *p* < 0.01; ***, *p* < 0.001). ^4^ NS, not significantly different.

## Supplementary Materials

The following are available online: Figure S1: Schematic diagram of this study.
