# Supplementary material for: Effect of Fungi on Metabolite Changes in Kimchi During Fermentation
Source: Molecules. 2020 Oct 30;25(21):5040. doi: 10.3390/molecules25215040 (PMC7663158; doi:10.3390/molecules25215040)
Supplement: Supplementary file 1 [file molecules-25-05040-s001.pdf]

Effect of fungi on metabolite changes in kimchi during fermentation

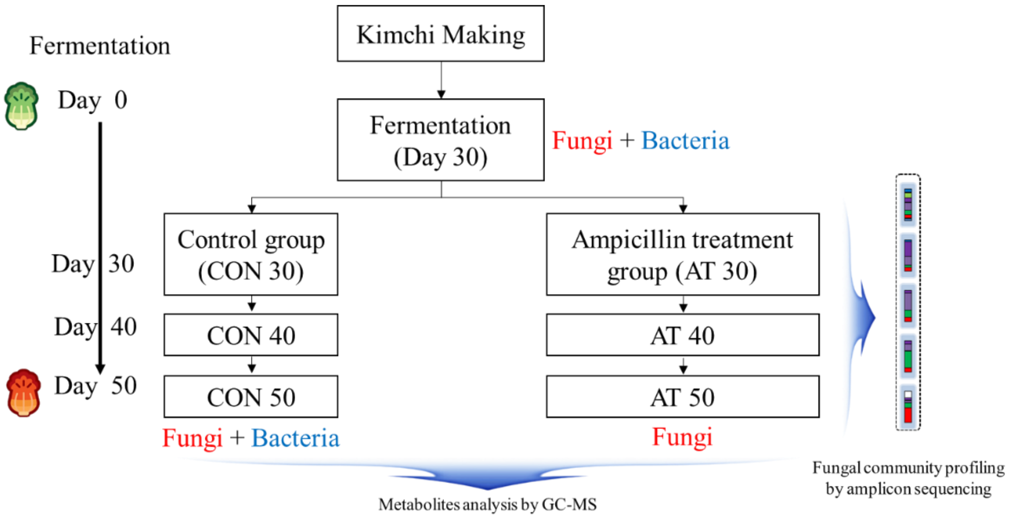

Based on the pattern of change of each metabolite, it was grouped into four categories

1) If CON 30 ≠ CON 50 and CON 30 ≠ AT 50 and AT 50 = CON 50 → Fungi related metabolites
2) If CON 30 ≠ CON 50 and CON 30 ≠ AT 50 and AT 50 ≠ CON 50 → Fungi + Bacteria related metabolites
3) If CON 30 ≠ CON 50 and CON 30 = AT 50 and AT 50 ≠ CON 50 → Bacteria related metabolites
4) If CON 30 = CON 50 and CON 30 ≠ AT 50 and AT 50 ≠ CON 50 → Undetermined

Symbols (=) and (≠) indicate significantly different and not significantly different, respectively.

Figure S1: Schematic diagram of this study.
